# Complications of Pelvic Prolapse Surgery Using Mesh: A Systematic Review

**DOI:** 10.3390/jpm14060622

**Published:** 2024-06-11

**Authors:** Alexandru Dabica, Oana Balint, Flavius Olaru, Cristina Secosan, Ligia Balulescu, Simona Brasoveanu, Marilena Pirtea, Diana Popin, Ioana Flavia Bacila, Laurentiu Pirtea

**Affiliations:** 1 Doctoral School, Victor Babes, University of Medicine and Pharmacy, Eftimie Murgu Square No. 2, 300041 Timisoara, Romania; alexandru.dabica@umft.ro (A.D.); ligia.balulescu@umft.ro (L.B.); marilena.pirtea@umft.ro (M.P.); diana.popin@umft.ro (D.P.); ioana.bacila@umft.ro (I.F.B.); 2 Department of Obstetrics and Gynecology, Victor Babes University of Medicine and Pharmacy, 300041 Timisoara, Romania; olaru.flavius@umft.ro (F.O.); cristina.secosan@umft.ro (C.S.); simona.brasoveanu@umft.ro (S.B.); pirtea.laurentiu@umft.ro (L.P.)

**Keywords:** mesh complications, prolapse, vaginal, laparoscopic

## Abstract

Background: Pelvic organ prolapse (POP) is a public health problem that influences millions of women around the globe, and it has a significant impact on the quality of life. From the FDA statement regarding the complications of using mesh implants in POP surgery to studies that have shown the benefits and side effects, we conducted a systematic review investigating the complications associated with surgical mesh implantation for POP repair. Methods: Relevant studies were identified through a comprehensive search of scientific databases. Studies evaluating the use of mesh in POP surgery and reporting on associated complications were included. Results: Among 2816 studies, 28 studies met the research criteria, with a total number of 8958 patients, revealing that in laparoscopic mesh surgery, the rate of mesh exposure was lower compared to vaginal mesh surgery, among other complications. Conclusions: Laparoscopic mesh surgery is superior as a long-term approach for POP repair compared to vaginal mesh surgery, offering lower complication rates and potentially better anatomical success. However, vaginal mesh surgery remains a valuable option for patients who are unsuitable for laparoscopy due to specific factors. Future research should explore alternative techniques, like pectopexy with or without mesh, to further improve surgical outcomes and patient experience.

## 1. Introduction

Pelvic organ prolapse (POP) is defined as the descent of pelvic organs in the vagina from their anatomically correct positions. Major risk factors for developing POP include vaginal delivery, a higher body weight, and an advanced age. This condition can lead to complications affecting the bladder, bowel, and sexual function, significantly impacting an individual’s quality of life. Furthermore, the overall incidence of pelvic organ prolapse continues to demonstrate an upward trend, likely due to a combination of factors, including an aging population and the rising prevalence of obesity [1]. Several studies have documented a wide variability in the prevalence of pelvic organ prolapse, ranging from 3% to 50% [2]. Consistently, higher POP rates are observed in women with advanced ages and a history of multiple childbirths (Wang et al.). The incidence of POP reported globally in 2019 was estimated to be 13 million new cases [3].

It is essential to distinguish between the objective presentation of POP’s physical signs and an individual’s subjective experience of those signs. Treatment for POP should focus on alleviating the specific symptoms reported by the patient. Therefore, it is crucial to take into account the patient’s reported symptoms when formulating a diagnosis and treatment plan. 

Pelvic organ prolapse (POP) may necessitate surgical intervention in a subset of patients. However, data suggest that surgical management is undertaken by approximately 19% of women who are diagnosed with POP throughout their lifetimes. It is noteworthy that following surgical management, a recurrence of pelvic organ prolapse (POP) has been documented in 10% to 30% of patients, necessitating repeat surgery. Research experts predict a significant rise in pelvic organ prolapse surgeries, with estimates suggesting a 47% increase over the next 40 years [4].

Surgical options for POP treatment encompass classic abdominal surgery, laparoscopic surgery, robotic surgery, and vaginal surgery. Minimally invasive approaches, such as laparoscopic and robotically assisted laparoscopic sacrocolpopexy, are gaining prominence for pelvic organ prolapse surgery due to their advantages of shorter hospital stays, quicker recovery times, minimal blood loss, and reduced postoperative pain [5,6].

A history of previous surgery for POP is a risk factor for the condition’s recurrence. It has been reported that the anterior compartment is the most frequent site of prolapse, occurring twice as often as the posterior compartment and three times more frequently than the apical compartment defect [7]. 

Various surgical techniques employing synthetic mesh have been developed to treat POP. Research studies have shown that using mesh during surgery can reduce the likelihood of prolapse recurrence, although it may not necessarily improve the patient’s quality of life. Moreover, there are associated risks with using synthetic mesh, such as pain and exposure of the mesh [8].

The use of vaginal synthetic mesh in treating POP has been a subject of considerable debate due to conflicting evidence. The U.S. Food and Drug Administration (FDA) has reclassified synthetic mesh for POP repair to be under the most rigorous level of review. In April 2019, the FDA ordered manufacturers to cease the sale and distribution of synthetic mesh products for repairing anterior compartment prolapse due to concerns about their safety and effectiveness [9]. While it might be associated with a lower risk of POP recurrence in the short term, there is also a higher incidence of complications such as pelvic pain, mesh exposure, and extrusion [8].

Optimal surgical mesh for POP repair should possess two critical properties: ideal flexibility and ample strength. Flexibility allows for easier handling and positioning during surgery, potentially leading to a smoother surgical experience and improved outcomes. The mesh needs to be robust enough to provide long-term structural support for the pelvic organs, preventing them from prolapsing again [10]. The pores should be large enough to facilitate the ingrowth of fibroblasts, which play a vital role in tissue integration. Their presence helps the mesh integrate seamlessly with the surrounding tissues, potentially reducing the risks of mesh exposure and wearing through tissue and rejection by the body’s immune system [10].

While evidence suggests that mesh may be more effective than native tissue repair for pelvic organ prolapse (POP), it comes with higher risks of complications and repeat surgeries. Despite having promising initial success rates of 87% to 95%, mesh’s safety and long-term effectiveness are under scrutiny due to the reoperation rate of 6% and the high risk of exposure, namely 4.6–10.7% [11].

This study aimed to review the current evidence regarding mesh use and its complications.

## 2. Materials and Methods

We conducted searches on the PubMed and Scopus databases, emphasizing articles written only in English. The keywords used were mesh complications, prolapse, vaginal, laparoscopic, and terms associated with those. This systematic review has not been registered.

We chose an interval of 10 years, from 2014 to 2024, considering that from 2014 onward, research papers that debate mesh complications in vaginal and laparoscopic gynecologic surgery in the treatment of prolapse thrived the most.

**Inclusion criteria:**

1. Original articles such as clinical trials and randomized controlled trials;
2. Studies involving only humans;
3. Studies employing the treatment of genital prolapse only;
4. Studies written in English.

**Exclusion criteria:**

1. We excluded studies that were not available in full or not relevant according to the abstract;
2. We excluded articles that discuss mesh complications in fields of surgery other than gynecology;
3. We excluded other types of articles, like reviews, case reports, and conference abstracts;
4. We excluded studies written in languages other than English;
5. We excluded studies involving animals and in vitro experiments/in vivo experiments.

The methodological quality of the included studies was evaluated using the six-point criteria established by Hayden et al. [12]. These criteria encompass critical aspects of study design and execution, including sample adequacy and representativeness, which assesses whether the study population is sufficient and whether it appropriately reflects the target population of interest; **confounding variable management**, which evaluates the strategies employed within the study to mitigate the influence of extraneous variables that could impact the observed relationship between the exposure and the outcome; **factor of prognostic**, which assesses the clarity and comprehensiveness of the definition and description of the specific factor investigated in the study; **study attrition management**, which evaluates the extent to which the authors provide a detailed explanation for participant dropout and its potential impact on the study’s conclusions; **statistical analysis appropriateness**, which assesses whether the statistical methods employed within the study are aligned with the overall study design and the specific research question being addressed; and **outcome measurement rigor**, which evaluates the comprehensiveness of the explanation provided regarding the outcome measurement method. Specifically, it assesses the steps taken to minimize measurement bias. Each criterion was assigned a score ranging from 0 to 3 points. Studies scoring between 0 and 3 points were classified as low quality, while those scoring above 3 and up to 6 points were considered high quality.

**Figure 1:**
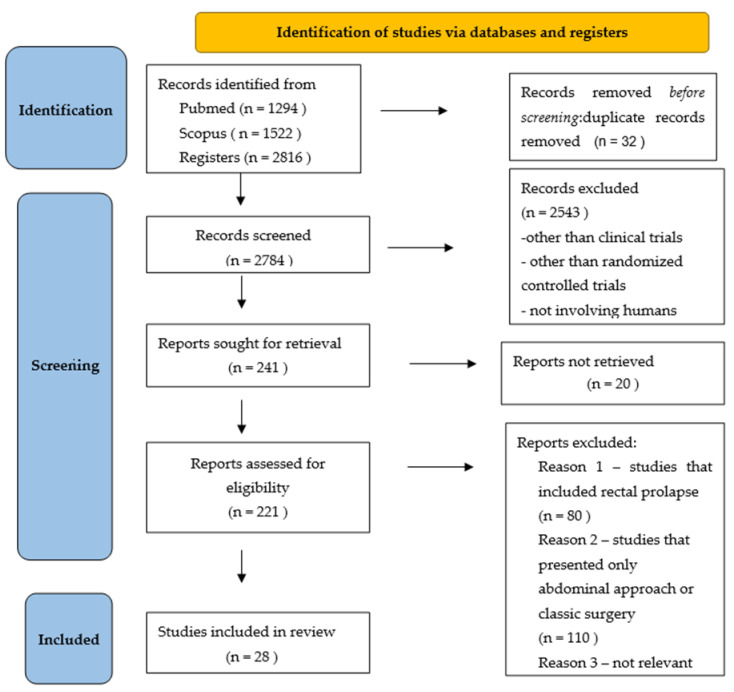
PRISMA chart.

A total of 2816 articles were identified, with 2784 being screened after removing duplicates (Figure 1). After applying the inclusion and exclusion criteria, 241 articles remained. The number of excluded articles was 2543; these included studies other than clinical trials and randomized controlled trials, did not involve human subjects, or were not written in English. Twenty articles were not retrieved. We obtained 221 reports, which were assessed for eligibility. We excluded studies that included rectal prolapse, studies that only presented an abdominal approach or classic surgery, and two studies that were not relevant; in one study, the results were missing from the article. We included 28 studies’ full texts in the review, with a total of 8958 patients. Articles were dated from 2014 to 2024. There were 24 randomized controlled trials, 5 prospective cohort studies, 1 retrospective study, 1 non-randomized controlled trial, and 1 observational cohort study.

## 3. Results

### 3.1. Publication Demographics

**Figure 2:**
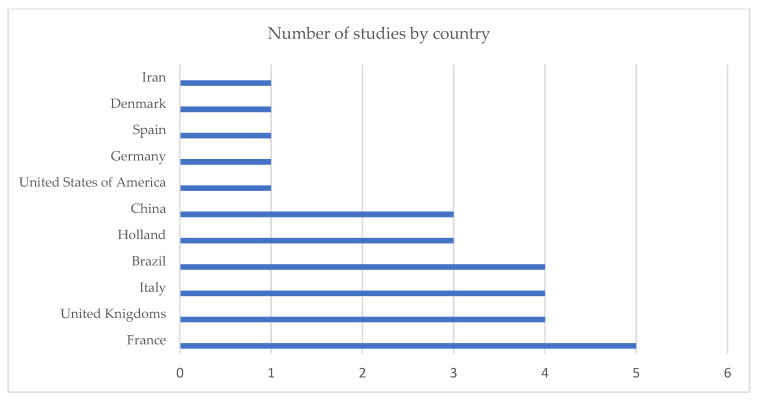
Publication demographics.

The studies took place in 11 countries (Figure 2). France (17.8%) was the country with the most significant number of studies [13,14,15,16,17], followed by the United Kingdom (14.3%) [18,19,20,21], Italy (14.3%) [7,22,23,24], Brazil (14.3%) [25,26,27,28], Holland (10.8%) [29,30,31], China (10.8%) [32,33,34], the United States of America (3.6%) [35], Germany (3.6%) [36], Spain (3.6%) [8], Denmark (3.6%) [37], and Iran (3.6%) [38].

### 3.2. Number and Design of Studies

In this review, we included 28 studies (Table 1). The predominant study design among the eligible studies was clinical randomized trials. Upon evaluating the methodological quality of the included studies, it was found that 20 studies attained a score of 6 points, 4 studies scored 5 points, and 4 studies scored 3 points.

### 3.3. Patients Number

The studies included a varied number of patients, ranging from 32 to 2309 individuals. In the context of prolapse surgeries, the laparoscopic surgery group encompassed 16 to 1113 patients, while the vaginal surgery group involved 16 to 692 patients.

### 3.4. Number of Laparoscopic, Vaginal, and Mixed Studies

We included 7 studies [7,8,13,22,23,35,36] that only involved laparoscopic treatment, 3 studies regarding transvaginal mesh treatment [17,33,34], and 18 mixed studies (mesh laparoscopic and vaginal mesh, vaginal mesh and native tissue, biologic xenograft and vaginal mesh, laparoscopic mesh and robotic mesh, and laparoscopic mesh and abdominal mesh) [14,15,16,18,19,20,21,24,25,26,27,28,29,30,31,32,37,38].

### 3.5. The Anatomic Compartment of Prolapse Involved

The majority of the compartments involved in the studies were the anterior and apical compartments, followed by the posterior compartment (Table 2).

### 3.6. Mesh Type

The predominant synthetic material utilized in prolapse surgery is polypropylene mesh. Additional materials employed in these procedures include surgical glue with cyanoacrylate, monofilament sutures, polyester sutures, polyglactin sutures, and Gore-tex sutures. Notably, a specific mesh type was not specified in a particular study [15].

#### Light Meshes

In the context of light meshes, various types, such as titanized ultralight mesh, ultralight mesh, and light mesh, were utilized across five separate studies [7,17,22,32,35]. The findings indicate improved outcomes in relation to mesh exposure at 3 months, 1 year, and 2 years of follow-up (Table 3). It is noteworthy that three studies [22,32,35] reported zero instances of mesh exposure at the 1-year follow-up period subsequent to laparoscopic surgery for POP.

### 3.7. Main Outcome

The primary outcome was diverse in some studies, but the most cited outcome was anatomic success, defined as vaginal apex descent no more than 1/3 into the vagina and the anterior and posterior vaginal walls not descending beyond the hymen. Other outcomes included short- and mid-term feasibility, strength and tolerance of glue mesh, reoperation for apical prolapse, operative times, quality of life, and rate of surgical complications according to the modified Clavien–Dindo classification at one year. Additionally, the outcomes between laparoscopic sacral colpopexy with laparoscopic pectopexy were compared, with severe complications and subsequent reoperations for POP recurrence; anatomical outcomes were compared with subjective outcomes, with efficacy being measured as the difference in the anatomical recurrence rate of mesh versus anterior colporrhaphy, optimal surgical management, clinical and cost effectiveness, and adverse events; and the effects of preoperative vaginal estrogen and non-vaginal estrogen therapy on the incidence of mesh exposure, perioperative morbidity, and early complications were compared.

### 3.8. Second Outcomes

The second most cited outcomes were mesh exposure, reoperation rate, postoperative complications, and sexual function, followed by quality of life, the incidence of anatomical failures, the prevalence of urinary and anorectal symptoms, the efficiency of the new techniques, and the expected differences regarding defecation disorders. We also drew a comparison between the operative time and hospitalization costs.

### 3.9. Complications and Follow-Up

The following information outlines the key complications associated with mesh surgeries for prolapse. These include mesh exposure/extrusion, de novo urinary incontinence, dyspareunia or pelvic pain, and defecation difficulties (“bowel symptoms”) (Table 4). The terminology used in the studies, such as “mesh erosion”, has been re-evaluated by the International Urogynecological Association (IUGA) and the International Continence Society (ICS) to encourage the use of more specific and clinically relevant terms for enhanced clarity and precision. Specifically, “exposure” refers to the visualization of implanted mesh material within the vaginal cavity and a disruption of the overlying vaginal epithelium, while “extrusion” denotes the protrusion of the implanted material through the overlying tissue layers (example: a loop of surgical tape protruding into the vagina) [39]. It is worth noting that the incidence of complications was notably higher in vaginal surgery for prolapse compared to laparoscopic surgery, even with more extended follow-up periods. The follow-up ranged from 1 month to 110 months (9 years and 2 months). In most studies, the follow-up was settled at 1 year (last medical check-up). 

In terms of specific percentages, the incidence of mesh exposure in vaginal surgery ranged from 0.9% to 20%, the incidence of de novo urinary incontinence ranged from 3.3% to 25.6%, the incidence of dyspareunia ranged from 0.9% to 19%, and the incidence of defecation difficulties ranged from 1.8% to 6.6% at one year of follow-up. Conversely, in laparoscopic surgery, the percentages were as follows at one year of follow-up: mesh exposure (0%–6%), de novo urinary incontinence (3%–12%), dyspareunia (0%–14%), and defecation difficulties (0%–19.5%).

Furthermore, it is of significance to highlight four studies with more extended follow-up periods: one study had a follow-up at six years [19], two studies had a follow-up at seven years [18,31], and one study had a follow-up at nine years [30]. The incidence of mesh exposure was notably observed in the study with 74 patients and a follow-up of nine years and two months, reporting a rate of 18.8% [30]. In another study with 62 patients, namely 33 in the laparoscopic group and 29 in the vaginal group, with a seven-year follow-up, the authors did not report mesh exposure after using the laparoscopic technique [18]. Notably, in two studies that used vaginal surgery as a treatment method for genital prolapse, at six years of follow-up, with 432 patients, the rate of mesh exposure was 8,4%, and at seven years of follow-up, with 66 patients, the rate of mesh exposure was 42% [19,31]. In a 5-year follow-up study, with 122 patients including 63 patients in the transvaginal mesh group and 59 patients in the transvaginal native tissue group, there was a 27% mesh exposure rate [26].

### 3.10. Percentage of Complications Based on The Clavien-Dindo Classification

Six studies monitored the rate of complications classified as higher than III, which includes the necessity of surgical, radiological, or endoscopic interventions, based on the Clavien–Dindo classification (a classification of postoperative complications) [7,15,16,17,24,33]. Two studies that utilized lightweight meshes demonstrated a rate of Clavien–Dindo classification of 0.8% in laparoscopic surgery and 2.8% in vaginal surgery [7,17]. The percentage of complications in vaginal mesh surgery ranged from 1.2% to 9.37%, while in laparoscopic mesh surgery, it ranged from 0% to 0.8%. In the other twenty-two studies, the complication rate was not specified based on the Clavien–Dindo classification.

### 3.11. Rate of Anatomic Success

Thirteen studies evaluated the rate of anatomic success based on the follow-up depending on the study, namely at one year and two, four, seven, and nine years [14,17,18,24,27,28,29,30,31,32,33,34,35]. For laparoscopic mesh surgery (Table 4), at one year of follow-up [14,24,29,32,35], anatomic success ranged from 89.8% to 100%; at seven years, it was 94% [18]; and at nine years, it was 78.6% [30]. On the other hand, for vaginal mesh surgery (Table 5), anatomic success at the one-year follow-up ranged from 86.7% to 99.5% [29,32]; at two years, it ranged from 85% to 95.2% [27,28]; and at seven years, it ranged from 73% to 83% [18,31]. In the studies that used lightweight meshes that were specified, the rate of anatomic success ranged from 97.4% to 98.5% [32,35] for laparoscopic surgery, and that for vaginal surgery ranged from 86.7% to 87.9% [17,32]. In the other sixteen studies, the anatomic success rate was not specified.

### 3.12. Reintervention Rate

The reintervention rate was reported in eighteen studies, with variations depending on the type of surgery (laparoscopic or vaginal) and the duration of the follow-up period [7,8,13,14,15,16,18,20,21,25,26,29,30,31,32,35,36,38]. At one year of follow-up for laparoscopic mesh surgery (Table 5), the reintervention rate ranged from 0 to 9.8% [8,16,29,32,35,36,38]; at two years, it ranged from 6.7% to 9.52% [7,13]; at four years, it was 4.3% [14]; at seven years, it was 6.1% [18]; and at nine years, it was 22.7% [30]. For vaginal mesh surgery (Table 6), the rate of reinterventions ranged from 0% to 10.9% at one year of follow-up [16,20,25,29]; at two years, it was 3% [28]; at four years, it was 5.8% [14]; and at seven years, it ranged from 13% to 17.2% [18,31].

## 4. Discussion

The existing literature on the treatment of genital prolapse supports both laparoscopic surgery and vaginal surgery as viable approaches. A variety of techniques and materials are available for prolapse treatment, including laparoscopic sacropromontopexy/sacrocolpopexy with mesh, transvaginal mesh repair, pectopexy with wires, transvaginal repair with native tissue or xenograft, abdominal classic surgery, and robotic sacropromontopexy with mesh. In this review, we focused on the use of mesh in laparoscopic surgery and vaginal surgery. The majority of the patients presented with grade III or IV genital prolapse.

The anterior compartment was the most involved, as stated in the literature and in the studies we included in this review. 

Over the past 10–15 years, the use of surgical mesh has been linked to an elevated risk of complications. While acknowledging this and the budgetary constraints faced by healthcare systems globally, the potential for higher costs associated with lighter mesh materials must be carefully balanced against the established goal of minimizing the short- and long-term risks observed with currently implanted materials. This is particularly pertinent in developing or mid-development countries, including those in Europe, where assessing the cost-efficiency benefits and the impact on their medical systems may prove challenging.

In this review, at one year of follow-up, which was the period used by the majority of the studies, the incidence of mesh exposure in vaginal surgery ranged from 0.9% to 20%, while in laparoscopic surgery, it ranged from 0% to 6%. Cundiff et al. reported a 5% mesh exposure rate, on average, after abdominal sacrocolpopexy surgery, with a mean interval of 313 days from surgery to exposure [40]. Nygaard et al. found that the mesh exposure rate increased to 10.5% after six years of follow-up [41]. In one of the studies included in the review, with 74 patients and a follow-up of nine years and two months, the mesh exposure rate was 18.8% [30]. In another study with 62 patients including 33 in the laparoscopic group and 29 in the vaginal group, with seven years of follow-up, the authors did not report mesh exposure when using the laparoscopic technique [18]. Van Zanten et al., in a prospective study and an overview of the literature, reported 65 studies that described mesh exposure after laparoscopic sacrocolpopexy, with an exposure rate between 0 and 13.3%, and the range of number of patients included was 12–4552, with a range of follow-up of 12–72 months [42]. Eighty-three percent of the studies included in Van Zanten et al.’s overview reported an exposure rate lower than or equal to 5%. Most of the exposure rates reported in the studies were symptomatic [42]. 

Even if the mesh has a well-known complication, recent studies using ultralight polypropylene meshes reported decreasing mesh exposure. Two studies included in this review, which used lightweight and ultralightweight mesh, did not report mesh exposure at one year of follow-up after laparoscopic sacrocolpopexy [22,35]. Salamon et al. and Culligan et al., who kept evidence of 261 patients with one year of follow-up, also did not report mesh exposure after laparoscopic surgery when using ultralight and lightweight polypropylene meshes. Even if the exposure rates can increase in time, they might still be lower compared to those of traditional mesh [43,44].

An interesting approach was used by a study that we reviewed. Panel et al. used polypropylene mesh fixed by synthetic biodegradable surgical glue with cyanoacrylate in minimally invasive sacrocolpopexy, reporting a 2,38% mesh exposure rate at two years of follow-up [13]. Using glue fixation led to a significant prolapse improvement and an increased quality of life, and it did not lead to increased mid-term morbidity. This was the first multicenter prospective study on minimally invasive sacrocolpopexy using surgical glue for mesh [13].

Within the scope of this review, three studies focused on vaginal mesh surgery as a treatment for genital prolapse. With five, six, and seven years of follow-up, these studies reported mesh exposure ranging from 8.5% to 42% [19,26,31]. Additionally, Jacquetin et al. noted that over 50% of mesh-associated complications manifested within the first postoperative year in a study with a five-year follow-up after transvaginal mesh surgery [45]. Furthermore, Abed et al., in a systematic review involving 11,785 and 110 studies, underlined the FDA’s warnings about mesh and reported a mesh exposure incidence rate of 10.3% (range 0–29.7%) after vaginal mesh surgery in the initial postoperative year. The incidence rate for postmenopausal patients was higher [46]. Dandolu et al. confirmed this high incidence [47]. Halaska et al. reported an exposure rate of 15.6% at three months, which rose to 20.8% by one year [48]. Balzarro et al. reported an mesh exposure rate of 16.7% at five years of follow-up [49].

The consulted literature recommends the administration of vaginal estrogen cream for 4 to 6 weeks prior to prolapse surgery to reduce mesh exposure [50,51]. A study that was taken into consideration in this review demonstrated that in women with severe genital prolapse requiring transvaginal mesh surgery, vaginal estrogen therapy was not superior to non-vaginal estrogen therapy at one year of follow-up, and the mesh exposure rate was 14,5%. A possible explanation for this could be that mesh exposure is a complex mechanism correlated with graft properties, patients’ personal characteristics, the lengths of follow-up periods, and surgical skills, and it has also been a debate whether vaginal estrogen cream would thicken the walls of the vagina [34].

Synthetic materials offer a promising approach and potentially higher success rates in pelvic reconstructive surgery, but they come with a risk of complications other than mesh exposure. Careful consideration is needed before using these materials. The research by Altman et al. demonstrated that the mesh procedure achieved more tremendous short-term success than the traditional method. However, it was also associated with an increased incidence of complications such as bladder perforation and exposure [52].

The studies that we reviewed regarding transvaginal mesh surgery revealed an incidence range of 3.3–25.6% for de novo urinary incontinence, 0.9–19% for dyspareunia, and 1.8–6.6% for defecation difficulties at one year of follow-up compared to laparoscopic mesh surgery, which had a range of 3–12% for urinary incontinence, 0–14% for dyspareunia, and 0–19.5% for defecation difficulties also at one year of follow-up. Notably, except for defecation difficulties, the rates of mesh exposure, de novo urinary incontinence, and dyspareunia were lower for laparoscopic surgery. The anatomic success rates were comparable during the first year after surgery; however, at seven years of follow-up, laparoscopic surgery demonstrated a superior anatomic success rate of 94% compared to 78% [18,31].

Recent publications have highlighted that a preoperative diagnosis of stage III or higher pelvic organ prolapse constitutes a risk factor for postoperative recurrence when native tissue repair is employed [8]. In three studies, namely those by Frittel et al., Hemming et al., and da Silveira et al., a higher rate of recurrence of native tissue repair was reported (2.8–24.7%) compared to that of transvaginal mesh repair [15,20,26]. Many urogynecologists recognize vaginal hysterectomy (VH) with apical suspension as a preferred surgical approach for uterine prolapse. This preference acknowledges the association between the frequently occurring anterior and apical prolapse [53]. Jelovsek et al. revealed that vault prolapse post-hysterectomy has a risk rate between 4.6 and 18% at five years of follow-up [54].

According to both a literature review [55] and a meta-analysis of pertinent studies [56], there is a statistically significant increase in the reintervention rates for patients who underwent vaginal vault suspension (VSF) compared to those who underwent sacrocolpopexy [55,56].

Our review noted that the reintervention rate within one year post-surgery is comparable between those of the laparoscopic sacral colpopexy (LSC) and transvaginal mesh (TVM) approaches, with rates ranging from 0% to 9.8% for LSC versus 0% to 10,9% for TVM. However, the TVM approach shows higher rates at the four-year and seven-year follow-up marks [14,18].

According to the Clavien–Dindo classification, vaginal mesh surgery exhibits a higher incidence of complications >3, ranging from 1.2% to 9.37%, in comparison to laparoscopic mesh surgery, which ranges from 0% to 0.8%.

Another topic worth mentioning is Shoenfeld’s syndrome or autoinflammatory/autoimmunity syndrome induced by adjuvants (ASIA). Cohen Tervaert JW conducted a prospective study with 700 patients referred to an autoimmunity clinic between January 2014 and December 2017, where 22 patients had a vaginal mesh implant and 18 patients underwent a hernia mesh repair. All 40 patients reported fatigue, myalgias, and other general symptoms. Notably, nearly all patients had a history of allergies [57]. Additionally, half of the patients developed an autoimmune disease, and a quarter showed signs of immunodeficiency. The conclusion was that 40 patients developed a systemic illness following mesh implantation. The authors propose that the mesh may have triggered this illness by activating the immune system in susceptible individuals [57]. In a systematic review, Kowalik et al. did not identify a definitive link between mesh implantation for pelvic organ prolapse and the development of autoimmune diseases [58]. The prevalence of autoimmune diseases in the general population is quite variable, with estimated rates of 3.2% to 9.4% [59]. Chughtai et al. and Muller et al. reported that among all of the mesh groups, the one used for stress urinary incontinence (SUI) had the highest rate of autoimmune diseases (8.1%) [60,61]. The rates were much lower for the other mesh groups, namely 1.5% for hernia repair and 2.8% for pelvic organ prolapse repair [60,61]. The results are similar to those of Clancy et al., who did not find an association between mesh implantation and systemic autoimmune syndromes [9].

A limitation of this study is the heterogeneity in surgical techniques; even within laparoscopic or vaginal approaches, there might be variations in surgical techniques, mesh materials, and fixation methods, and sometimes, this heterogeneity can make it difficult to isolate the specific effects of laparoscopic compared to vaginal access. The generalizability of the conclusions derived from this research may be limited due to the evolution of materials employed in recent years, including the meshes used in the studies that we analyzed being different from those currently available.

A more significant number of studies that used lightweight meshes could be studied in order to demonstrate a better perspective and the frequent use of these types of meshes. Studies conducted in high-volume centers with experienced surgeons may achieve better outcomes than those in low-volume settings, limiting the generalizability of real-world practices. Only some of the studies evaluated the anatomical success rates, and this can limit the ability to make a complete comparison between the two approaches. Some outcome measures, like pain or quality of life, can be subjective and prone to reporting bias. Using validated and standardized outcome measures can help mitigate this issue. Even if we had six studies with follow-ups longer than two years, long-term complications and mesh durability might not be fully captured in studies with short follow-up periods.

Further research is needed because new and evolving laparoscopic techniques might need to be better represented in the available literature, potentially impacting the generalizability of the conclusions.

## 5. Conclusions

Both surgical approaches are efficient, substantially improve patient symptoms, and have similar success rates in the first year. When analyzing 5851 patients treated with mesh, regarding the rates of mesh exposure, de novo urinary incontinence, dyspareunia, reintervention, and the Clavien–Dindo classification, at any period of follow-up, it was found that laparoscopic mesh surgery had lower rates compared to vaginal mesh surgery, and even the anatomic success rate at seven years of follow-up had superior rates, demonstrating that it is a better approach. However, for select patients, transvaginal mesh surgery can serve as a viable option, particularly in cases where laparoscopy is deemed unsuitable due to factors such as obesity, intra-abdominal adhesions, or prior abdominal surgery. It is imperative to emphasize the necessity for new studies featuring extended follow-up periods. While mesh remains a viable treatment option, alternative techniques such as mesh pectopexy or wire pectopexy without mesh may offer superior outcomes regarding intra-operative efficiency, complication rates, the duration of hospitalization, and overall results.

## Figures and Tables

**Table 1:** The designs of the studies.

Randomized Controlled Trials	Non-Randomized Controlled Trial	Prospective Cohort Study	Retrospective Study	Observational Cohort Study
24 studies	1 study	5 studies	1 study	1 study

**Table 2:** Anatomic compartment involved.

Anterior Compartment	Apical Compartment	Mixed Compartment (Anterior and Apical; Anterior, Apical, and Posterior)
5 studies	4 studies	19 studies

**Table 3:** Mesh exposure rates in laparoscopic surgery at 1-year and 2-year follow-ups and in vaginal surgery at 3-month and 1-year follow-ups.

Laparoscopic Surgery		Vaginal Surgery	
Follow-up	Mesh exposure	Follow-up	Mesh exposure
1 year	0%	3 months	2.8%
2 years	0.8%	1 year	3.3%

**Table 4:** Rate of complications in vaginal surgery and laparoscopic surgery at 1 year follow-up.

Vaginal Surgery		Laparoscopic Surgery	
Follow-up	1 year	Follow-up	1 year
Mesh exposure	0.9–20%	Mesh exposure	0–6%
De novo urinary incontinence	3.3–25.6%	De novo urinary incontinence	3–12%
Dyspareunia	0.9–19%	Dyspareunia	0–14%
Defecation difficulties	1.8–6.6%	Defecation difficulties	0–19.5%

**Table 5:** Rate of anatomic success in laparoscopic surgery at 1 year, 7 years, and 9 years of follow-up and rate of anatomic success in vaginal surgery at 1 year, 2 years, and 7 years of follow-up.

Laparoscopic Surgery		Vaginal Surgery	
1 year	89.8–100%	1 year	86.7–99.5%
7 years	94%	2 years	85–95.2%
9 years	78.6%	7 years	73–83%

**Table 6:** Rate of reintervention in laparoscopic mesh surgery at 1, 2, 4, 7, and 9 years of follow-up and rate of reintervention in vaginal surgery at 1, 2, 4, and 7 years of follow-up.

Laparoscopic Mesh Surgery		Vaginal Mesh Surgery	
1 year	0–9.8%	1 year	0–10.9%
2 years	6.7–9.52%	2 years	3%
4 years	4.3%	4 years	5.8%
7 years	6.1%	7 years	13–17.2%
9 years	22.7%		

## Data Availability

The data are contained within the article.
